# Gender and triptan efficacy: a pooled analysis of three double-blind, randomized, crossover, multicenter, Italian studies comparing frovatriptan vs. other triptans

**DOI:** 10.1007/s10072-014-1750-4

**Published:** 2014-05-28

**Authors:** Flavia Franconi, Cinzia Finocchi, Gianni Allais, Stefano Omboni, Vincenzo Tullo, Ilaria Campesi, Giorgio Reggiardo, Chiara Benedetto, Gennaro Bussone

**Affiliations:** 1 Laboratory of Gender Medicine, National Institute of Biostructures and Biosystems and Department of Neurological Sciences, University of Sassari, Via Muroni, 23, 07100 Sassari, Italy; 2 Department of Neurological Sciences, Ophthalmology and Genetics, University of Genoa, Genoa, Italy; 3 Women’s Headache Center, Department of Surgical Sciences, University of Turin, Turin, Italy; 4 Clinical Research Unit, Italian Institute of Telemedicine, Varese, Italy; 5 Department of Clinical Neuroscience, National Neurological Institute Carlo Besta, Milan, Italy; 6 Mediservice, Milan, Italy

**Keywords:** Migraine, Gender, Frovatriptan, Rizatriptan, Zolmitriptan, Almotriptan

## Abstract

Migraine is three times as common in females as in males, and attacks may be more severe and difficult to treat in women. However, no study specifically addressed possible gender differences in response to antimigraine therapy. The objective of this study was to review the efficacy of frovatriptan vs. other triptans, in the acute treatment of migraine in subgroups of subjects classified according to gender (men vs. women) through a pooled analysis of three individual randomized Italian studies. 414 patients suffering from migraine with or without aura were randomized to frovatriptan 2.5 mg or rizatriptan 10 mg (study 1), frovatriptan 2.5 mg or zolmitriptan 2.5 mg (study 2), frovatriptan 2.5 mg or almotriptan 12.5 mg (study 3). All studies had a multicenter, randomized, double-blind, crossover design. After treating 1–3 episodes of migraine in no more than 3 months with the first treatment, patients switched to the other treatment for the next 3 months. In this analysis, traditional migraine endpoints were compared between the 66 men and 280 women of the intent-to-treat population. At baseline, long-term and debilitating migraine attacks were more frequently reported by women than men. During the observation period, the proportion of pain-free attacks at 2 h did not significantly differ between frovatriptan and the comparators in either men (32 vs. 38 %, *p* = NS) or women (30 vs. 33 %, *p* = NS). Pain relief was also similar between treatments for both genders (men: 56 % frovatriptan vs. 57 % comparators; women: 55 vs. 57 %; *p* = NS for both). The rate of relapse was significantly lower with frovatriptan than with the comparators in men (24 h: 10 vs. 30 %; 48 h: 21 vs. 39 %; *p* < 0.05) as well as in women (24 h: 14 vs. 23 %; 48 h: 28 vs. 40 %; *p* < 0.05). The rate of adverse drug reactions was significantly larger with comparators, irrespectively of gender. Although migraine presents in a more severe form in women, frovatriptan seems to retain its good efficacy and favorable sustained antimigraine effect regardless of the gender.

## Introduction

Migraine is a chronic neurovascular disorder occurring in both genders, although large surveys show higher prevalence of this condition in women, with a female to male ratio in the order of 3:1 [1–4].

Although migraine acknowledges a complex pathophysiology, involving genetic and psychological factors [5], the disproportionate number of fertile women with migraine suggests that hormonal factors may indeed play an important role in the pathogenesis of migraine [6]. As a matter of fact, from adolescence migraine attacks are generally more common in women than in men, peaking during their 30 and 40 s, followed by a decline, particularly after menopause [7].This difference in migraine prevalence over a life time is mediated by the physiological fluctuation of estrogen level and consequently by its influence on cerebral vasculature, in women [8]. In addition, attacks are usually reported to be more severe and difficult to treat in women than in men [8].

The triptans, selective serotonin 5-HT_1B/1D_ receptor agonists, are very effective acute migraine drugs; they are currently recommended as a first-line treatment for moderate to severe migraine, or for mild to moderate migraine that has not responded to adequate doses of simple analgesics [9–11]. Frovatriptan is an antimigraine agent of the triptan class, developed to provide a triptan with a clinical potential for a long duration of action and a low likelihood of side effects and drug interactions [12, 13]. Three direct comparative, prospective, double-blind, randomized, crossover studies have recently compared the efficacy and safety of frovatriptan with that of rizatriptan [14], zolmitriptan [15], and almotriptan [16]. The study showed a similar efficacy of the four triptans in the immediate treatment of migraine, but lower recurrence rates, and thus a better sustained relief, with frovatriptan. Retrospective analyses of the same studies proved the good efficacy of frovatriptan also in subgroups of female migraine patients, such as those with menstrually related migraine [17] or with oral-contraceptive menstrual migraine [18].

Although there is no reason to doubt that current drug options for migraine treatment should display a similar efficacy in male and female migraineurs, so far no study specifically addressed possible gender differences in response to triptan therapy. To this purpose, in the present paper, we report on results of a pooled analysis performed in subgroups of migraineurs classified according to gender (males vs. females) and enrolled in previous direct comparative studies of frovatriptan vs. other triptans.

## Methods

### Study population and design

This pooled analysis is based on the data from three studies sharing a similar design and whose details are extensively reported in the original publications [14–16]. Overall, the studies included subjects of both genders, aged 18–65 years, with a current history of migraine with or without aura, according to International Headache Society (IHS) criteria, and with at least one, but no more than six migraine attacks per month for 6 months prior to entering the study. In this retrospective analysis, patients were classified in two subgroups according to gender (men and women).

The studies had a multicenter, randomized, double-blind, crossover design. Each patient received frovatriptan 2.5 mg or rizatriptan 10 mg [14], frovatriptan 2.5 mg or zolmitriptan 2.5 mg [15], frovatriptan 2.5 mg or almotriptan 12.5 mg [16] in a balanced computer-generated randomized sequence (1:1), where frovatriptan had to be followed by the comparator or vice versa. After treating 1–3 episodes of migraine in no more than 3 months with the first treatment, the patient had to switch to the other treatment. Subjects were encouraged to treat 1–3 attacks for a maximum period of 3 months with each study drug and to visit the center three times during the study. Subjects having no migraine episodes during one of the two observation periods were excluded from the study.

### Data analysis

In this analysis, traditional migraine endpoints were compared between men and women of the intent-to-treat population, defined as all patients treating at least one attack in each treatment period. The study endpoints were qualified according to International Headache Society Guidelines [19] as: (a) the number of pain free episodes at 2 h (absence of migraine episodes at 2 h after the intake of one dose of study drug and without any rescue medication); (b) the number of pain relief episodes at 2 h (defined as a decrease in migraine intensity from severe or moderate to mild or none, after the intake of one study drug dose); (c) relapse after 24 h (namely an episode which is pain free at 2 h and headache of any severity returns within 24 h, or requires the use of rescue medication or a second dose of study drug); (d) relapse after 48 h (namely an episode which is pain free at 2 h and headache of any severity returns within 48 h, or requires the use of rescue medication or a second dose of study drug). Safety analysis was applied to the intent-to-treat population, by calculating the incidence of adverse events during the study. Continuous variables were summarized by computing average values and standard deviation (SD), while categorical variables by computing the absolute value and the frequency (as percentage). Study endpoints were separately assessed according to gender (men vs. women) and compared between attacks treated with frovatriptan and with the comparators by analysis of variance (ANOVA), in case of continuous variables, and by Chi-squared test, in case of discrete variables. A subgroup analysis was carried out in postmenopausal and fertile women. All tests were two-sided and the level of statistical significance was kept at 0.05 throughout the whole study.

## Results

### Demographic and migraine feature of the study population

**Fig. 1:**
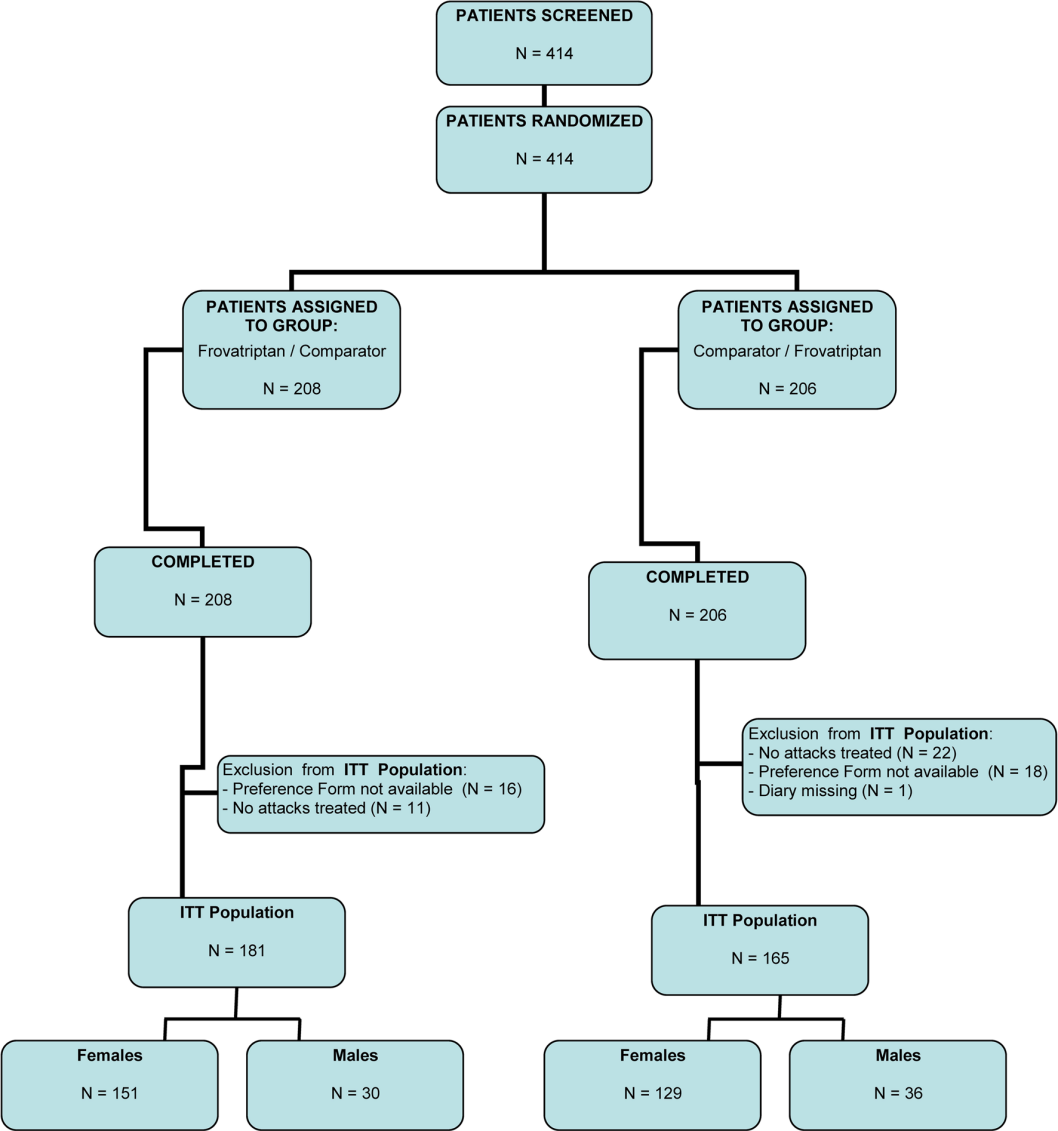
Flow diagram of the patients throughout the study

The intent-to-treat population consisted of 346 subjects, of which 66 (19 %) were men and 280 (81 %) women. A flow diagram of participants throughout the study is summarized in Fig. 1. Table 1 summarizes the main demographic and clinical characteristics of the intent-to-treat population at baseline, according to gender. The two study subgroups differed in several features at baseline, and particularly in those related to migraine severity. Women were younger, thinner and shorter than men. They reported their first attack earlier than men and the episodes were longer lasting and more debilitating (higher MIDAS score). Baseline intensity showed a statistically significant difference in the distribution of mild-moderate attacks between men and women, with attacks in women being more intense than in men.

**Table 1:** Demographic and clinical data of women and men and of postmenopausal and fertile women of the intent-to-treat population at the time of randomization

	Men (*n* = 66)	Women (*n* = 280)	*p* value	Postmenopausal women (*n* = 56)	Fertile women (*n* = 224)	*p* value
Age (years, mean ± SD)	40 ± 10	38 ± 6	<0.05	52 ± 5	34 ± 8	<0.0001
Height (cm, mean ± SD)	178 ± 7	163 ± 7	<0.0001	161 ± 8	164 ± 5	<0.001
Weight (kg, mean ± SD)	78 ± 13	59 ± 10	<0.0001	60 ± 10	59 ± 10	NS
BMI (kg/m^2^, mean ± SD)	25 ± 3	22 ± 4	<0.0001	23 ± 4	22 ± 4	<0.05
Age at onset of migraine (years, mean ± SD)	20 ± 10	18 ± 7	<0.05	22 ± 11	17 ± 6	<0.01
Migraine attack duration >2 days (*n*, %)	4 (6)	68 (24)	<0.01	16 (29)	52 (23)	NS
Migraine attacks with aura (*n*, %)	32 (5)	102 (8)	NS	39 (11)	67 (5)	<0.0001
MIDAS score (mean ± SD)	19 ± 15	23 ± 18	<0.05	25 ± 15	22 ± 19	NS
Baseline migraine severity (*n*, %)^a^						
Mild	106 (28)	303 (19)	<0.001	68 (17)	255 (21)	NS
Moderate	192 (51)	954 (60)		249 (62)	698 (58)	
Severe	82 (22)	346 (22)		85 (21)	248 (21)	
No use of triptans in the previous 3 months (*n*, %)	18 (27)	118 (42)	NS	22 (39)	96 (43)	NS

Data are shown as mean (±SD), or absolute (*n*) and relative frequency (%)

*BMI* body mass index, *MIDAS* migraine disability assessment

^a^Numbers refer to number and frequency of attacks as respect to overall number of attacks

Of the 280 women, 56 were postmenopause and 224 in fertile age. As expected, postmenopausal women were older than fertile women (Table 1). They also reported a higher rate of migraine attacks with aura and an older age at onset of the first migraine attack.

### Treatment efficacy of migraine attacks

During the observation period, a total of 1,978 attacks were recorded in the 346 migraineurs of the intent-to-treat population. Of these attacks, 331 (17 %) occurred in men and 1,647 (83 %) in women, 987 were treated with frovatriptan and 991 with comparators.

**Fig. 2:**
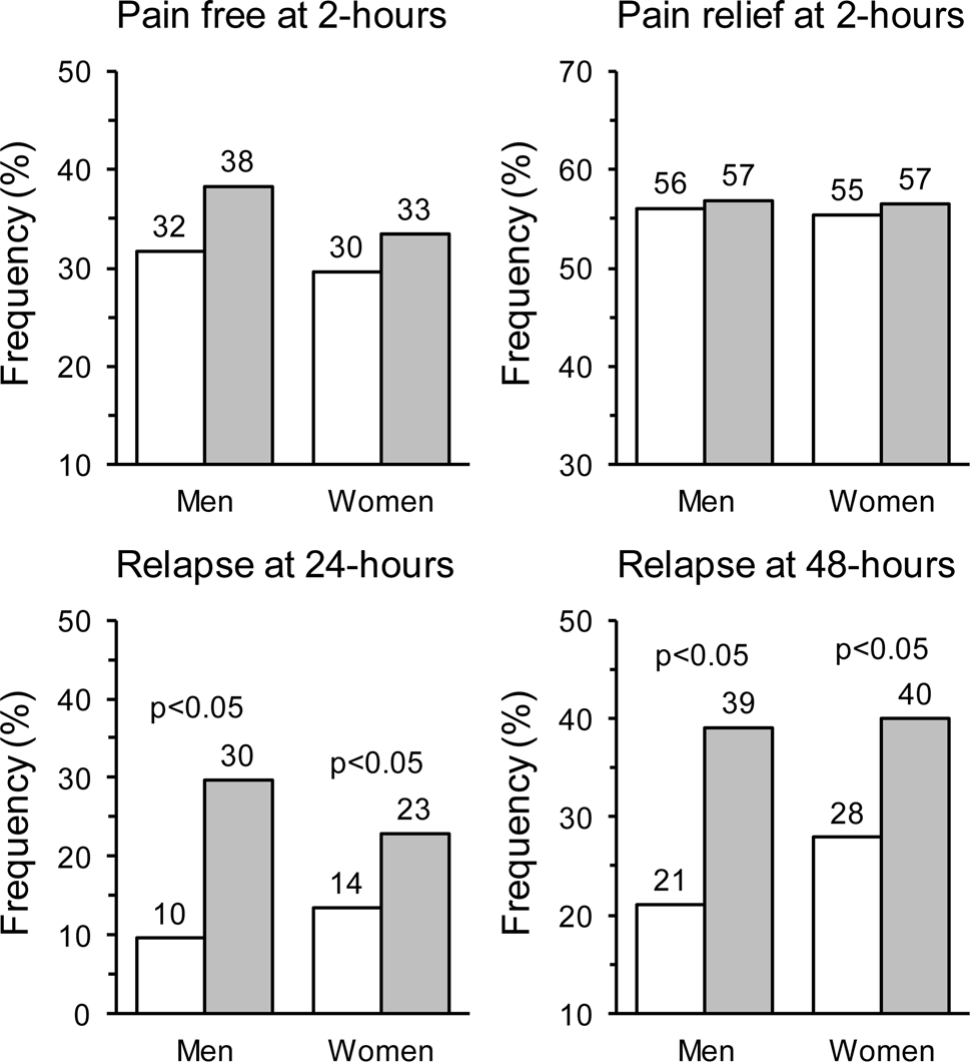
Proportion (%) of pain free at 2 h, pain relief at 2 h and relapse at 24 and 48 h in the 66 men and 280 women with migraine of the intent-to-treat population. Data are separately shown for frovatriptan- (*white bars*) and comparator-treated patients (*gray bars*). The *p* value refers to the statistical significance of the between-treatment difference

As shown in Fig. 2, at 2-h rate of pain-free episodes was not significantly different between frovatriptan and comparators, either in men (32 vs. 38 %; *p* = NS) or women (30 vs. 33 %; *p* = NS). Pain relief episodes at 2 h were also similarly distributed between the two treatments and both genders (men: 56 % frovatriptan vs. 57 % comparators; women: 55 vs. 57 %; *p* = NS for both). Conversely, relapse at 24 h was significantly (*p* < 0.05) less likely to be reported in frovatriptan than in comparator-treated patients, with no between-gender difference (men: 10 vs. 30 %; women: 14 vs. 23 %). This was the case also for rate of relapse after 48 h (men: 21 vs. 39 % and women: 28 vs. 40 %, *p* < 0.05 between treatments).

No statistically significant differences were ever observed between men and women in response to study treatments, for all the considered endpoints.

The efficacy of frovatriptan and comparators on pain free and pain relief at 2 h was identical in the subgroup of postmenopausal and fertile women (Table 2). However, the risk of relapse at 24 and 48 h was higher in women treated with the comparators, with a statistically significant between-treatment difference for women of fertile age.

**Table 2:** Study endpoints in men and subgroups of postmenopausal and fertile women of the intent-to-treat population treated with frovatriptan or the comparators

	Men (*n* = 66)			Postmenopausal women (*n* = 56)			Fertile women (*n* = 224)		
	Frovatriptan	Comparators	*p* value	Frovatriptan	Comparators	*p* value	Frovatriptan	Comparators	*p* value
Pain free at 2 h (%)	52/164 (32)	64/167 (38)	NS	56/173 (32)	64/180 (36)	NS	188/650 (29)	211/644 (33)	NS
Pain relief at 2 h (%)	62/123 (56)	70/123 (57)	NS	76/137 (56)	82/148 (55)	NS	294/531 (55)	300/528 (57)	NS
Relapse at 24 h (%)	5/52 (10)	19/64 (30)	<0.05	9/56 (16)	18/64 (28)	NS	24/188 (13)	45/211 (21)	<0.05
Relapse at 48 h (%)	11/52 (21)	25/64 (39)	<0.05	20/56 (36)	24/64 (38)	NS	48/188 (26)	86/211 (41)	<0.01

Data are shown as absolute values (number of attacks with the event and total number of attacks evaluated) and relative frequencies (%)

### Safety

A total of 133 adverse events were recorded in 2,033 treated attacks in the safety population (1,088 under frovatriptan and 945 under the other triptans), of which 55 occurred in men and 78 in women. The proportion of subjects with an adverse event was significantly (*p* < 0.05) lower with frovatriptan than with the comparators in both men (2 vs. 7 %) or in women (6 vs. 9 %).

## Discussion

In the present pooled analysis of three double-blind, randomized, crossover studies, acute treatment of male and female migraineurs with frovatriptan and with other triptans (rizatriptan, zolmitriptan and almotriptan) resulted in similar proportions of pain-free and pain relief episodes at 2 h, with no between-gender differences. However, frovatriptan showed a more sustained effect on relief of migraine symptoms than comparators, an effect that did not differ between men and women and such a finding is clinically relevant because it has been shown that female patients have a twofold greater risk than male patients of experiencing headache return following pain-free response [20]. The particular pharmacokinetics of frovatriptan, characterizing its slower onset of action and longer duration as compared to other triptans, may explain its greater efficacy in preventing headache recurrence [12].

From adolescence women experience more frequent, long-lasting and more painful headaches as compared to men, and are potentially less responsive to specific migraine treatment than men [4, 7]. For instance, in a study of risk factors for headache recurrence after oral and subcutaneous sumatriptan, recurrence following oral sumatriptan was more frequent in female patients [21]. In our retrospective analysis, we showed for the first time that no difference in response to triptan treatment exists between the two genders. Notably, such a lack of difference occurred even despite the fact that women presented at enrolment with more severe migraine symptoms than men.

Our results obtained in women also confirm other retrospective analyses performed on the same pooled study sample. In 187 of the 346 women who treated at least one episode of menstrually related migraine, rate of recurrence was significantly lower with frovatriptan than with comparators, either after 24 or 48 h [17]. Frovatriptan showed a more sustained relieving effect on migraine, with lower headache relapses over 24 h and even more so over 48 h, also in a subgroup of 35 women with oral-contraceptive-induced menstrual migraine [18].

Our study also provides some additional matters for discussion. We replicated the evidence from previous large observational studies that the prevalence of migraine is much higher in women than in men, and that in the female gender attacks tend to present in a more severe and debilitating fashion than in men [1–4, 7]. The effect of frovatriptan on relapses was more consistent when the subgroup of fertile women was considered, suggesting that this more numerous group of migraine patients may particularly benefit from frovatriptan treatment. Notably, the tolerability profile of frovatriptan was better than that of the comparators, irrespective of the gender, adding a further positive feature to the good efficacy pattern of the tested drug.

Despite the interesting results, we must recognize the limitation of the post hoc nature of our analysis: we performed a retrospective analysis on a subgroup of patients which were not originally selected for a gender-related study. However, to our knowledge, our report stands as the first large comparative systematic analysis of head-to-head trials of frovatriptan vs. other triptans in male and female migraineurs. In addition, we did not evaluate plasma levels of the different triptans, which act as substrates for different enzymes (see Table 3): some of these enzymes (e.g. CYP3A4 and CYP2D6) are differently expressed in men and women [22] suggesting that plasma levels of the various types of triptans may differ in the two sexes [23–30]. We think that all these limitations of our study are at least partially contrasted by the large number of subjects and migraine attacks included in the analysis.

**Table 3:** Enzymes involved in triptan metabolism, reported in order of importance

Triptan	Enzyme involved in the metabolism
Almotriptan [23, 24]	MAO-A, CYP3A4, CYP2D6
Frovatriptan [25, 26]	CYP1A2
Rizatriptan [27, 28]	MAO-A
Zolmitriptan [29, 30]	CYP1A2, MAO-A

*MAO* monoamine oxidase, *CYP3A4* cytochrome P3A4, *CYP2D6* cytochrome P2D6, *CYP1A2* cytochrome P1A2

In conclusion, the results of our combined analysis of individual data of three double-blind, randomized, crossover trials provide strong evidence that, in both men and women, frovatriptan seems to offer the advantage of a lower risk of recurrence as compared to other triptans. Our results might be helpful to stimulate the design and implementation of larger direct comparative randomized clinical trials evaluating triptan efficacy separately in men and women suffering from migraine.
